# Efficacy and safety of indocyanine green tracer-guided lymph node dissection in minimally invasive radical gastrectomy for gastric cancer: A systematic review and meta-analysis

**DOI:** 10.3389/fonc.2022.884011

**Published:** 2022-08-05

**Authors:** Jixiang Zhao, Ke Li, Zikang Wang, Qingqing Ke, Jiapu Li, Yizhen Zhang, Xiaojiang Zhou, Yunzhi Zou, Conghua Song

**Affiliations:** ^1^ Department of Gastroenterology, The First Affiliated Hospital of Nanchang University, Nanchang, China; ^2^ Department of Surgical Oncology, Capital Medical University, Beijing, China; ^3^ Department of Gastroenterolog, The Second Affiliated Hospital of Zhengzhou University, Zhengzhou, China; ^4^ Department of Surgical Oncology, Sun Yat-sen University Cancer Center, Guangzhou, China; ^5^ Department of Gastroenterology, The Affiliated Hospital (Group) of Putian University, Putian, China

**Keywords:** indocyanine green, laparoscopic gastrectomy, gastric cancer, efficacy, safety

## Abstract

**Background:**

The implementation of indocyanine green (ICG) tracer-guided lymph node dissection is still in the preliminary stages of laparoscopic surgery, and its safety and efficacy for gastric cancer remain unclear.

**Methods:**

A systematic review was conducted in PubMed, Embase, Web of Science, the Cochrane Library, and Scopus to identify relevant subjects from inception to June 2022. The core indicators were the total number of harvested lymph nodes and the safety of the laparoscopic gastrectomy with ICG. A meta-analysis was performed to estimate the pooled weighted mean difference (WMD) and 95% confidence interval (CI).

**Results:**

Thirteen studies and 2,027 participants were included (642 for the ICG-group and 1,385 for the non-ICG group). The mean number of lymph nodes dissected in the ICG group was significantly greater than that in the non-ICG group (WMD = 6.24, 95% CI: 4.26 to 8.22, *P <*0.001). However, there was no significant difference in the mean number of positive lymph nodes dissected between the ICG and the non-ICG groups (WMD = 0.18, 95% CI: −0.70 to 1.07, *P* = 0.879). Additionally, ICG gastrectomy did not increase the risk in terms of the operative time, estimated blood loss, and postoperative complications.

**Conclusion:**

ICG tracer with favorable safety increases the number of harvested lymph nodes but not the number of positive lymph nodes in laparoscopic gastrectomy. More high-quality, large-sample-size randomized controlled trials are still needed to enhance this evidence.

## 1 Introduction

Gastric cancer is a common malignant tumor. Its mortality rate ranks third among all cancers, making it the third most common cause of cancer-related deaths, with 784,000 global deaths in 2018 (1). On average, there are 990,000 new cases of cancer each year, and about 738,000 deaths from stomach cancer (2). The poor remission rate in laparoscopic gastrectomy is always linked to lymph node metastasis. The lymph nodes mainly distributed along blood vessels determine the importance of dealing with blood vessels for laparoscopic radical gastric cancer surgery, so the precise positioning of lymph nodes is critical in laparoscopic radical gastrectomy (3). Surgical treatment is still the first-line approach to treatment to provide a cure for gastric cancer (4). Meanwhile, laparoscopic surgery has a good effect on the short-term treatment of gastric cancer and has become a standard treatment at present (5). Although some progress in laparoscopic gastrectomy has been made in recent years, laparoscopic gastrectomy and lymph node dissection are still difficult to perform because of the complex anatomic structure and stomach vessel distribution (6, 7). Therefore, how to perform laparoscopic radical gastrectomy safely, effectively, and accurately is the focus of attention for researchers.

Indocyanine green (ICG) is being used as a new tracer agent in many malignant tumor surgeries (8). It fluoresced after the stimulus using a laser beam of 820 nm, or near-infrared light (NIR) (9). ICG has the characteristic of lymph orientation. ICG is injected into the serous membrane or submucosa (9). It will gather in the lymph nodes along with lymphatic vessels (9). It has been reported that the infrared imaging system can easily distinguish lymph nodes containing ICG from surrounding tissues due to fine tissue penetration of the signal of ICG (9, 10). A study found that the use of ICG demonstrates a higher sensitivity and specificity for sentinel lymph node mapping than other tracers (e.g., methylene blue, nanocarbon) (11). In recent years, indocyanine green has been used for non-invasive detection of lymphatic vessels and can show lymph nodes more clearly, which provides a new perspective for lymph node dissection (12). In other aspects, ICG has achieved good results in the lymph node dissection for endometrial carcinoma and colon cancer (13, 14). Since the development of the technique in recent years, Indocyanine green, a tracer for laparoscopic gastrectomy, has been widely used (15).

Sentinel lymph node biopsy guided by optical imaging combined with ICG is a good clinical diagnostic method, particularly for early gastric cancer (16). In recent years, the application value of ICG fluorescence imaging in lymph node dissection has become a new direction of exploration (17). ICG lymph node localization can improve the efficiency of lymph node dissection for advanced gastric cancer (17). The identification of small lymph nodes and dissection of some lymph node stations are its unique advantages (18).

However, the use of ICG during laparoscopic gastrectomy is still in its preliminary phase. Whether ICG can improve the lymph node detection rate and safety in laparoscopic gastrectomy compared with that in non-ICG laparoscopic gastrectomy is still controversial (19). Some studies have shown that the amount of fluorescent lymph node dissection in the ICG group is higher than that in the non-ICG group (20). But some articles said that it is uncertain whether fluorescence lymphography can be used for lymph node dissection (21). However, some studies indicated that there was no difference between the ICG and non-ICG groups regarding safety (20). ICG, a lymphatic tracer with minimal toxicity and few adverse effects, is a promising aid for achieving systematic and sufficient lymph node dissection. Clinical trials have been conducted to evaluate the value of ICG in laparoscopic radical gastrectomy. Although this intervention is potentially associated with benefits, available research is mostly confounded by small trials, uncontrolled reports or qualitative studies. The value of ICG has been the subject of several reviews (11, 22, 23). Since the publication of this meta-analysis, several high-quality studies (24, 25) were reported in 2018 and 2021, which could effectively increase the quality of this meta-analysis. Therefore, a systematic review specifically looking at evaluating the role of ICG in lymph node dissection during minimally invasive GC surgery, including all available research, was required to guide practice and future research.

## 2 Materials and methods

The Preferred Reporting Items for Systematic Reviews and Meta-Analyses (PRISMA) Reporting Guidelines (26) were used in this systematic review and meta-analysis to retrieve literature correlated with the efficacy and safety of ICG in laparoscopic gastrectomy. The literature was assessed by the Newcastal-Ottawa scale and the results are shown in **Table 1**.

**Table 1:** Details on included papers.

Year-Author	Country	Participants		Intervention		Outcomes						Study design	Newcastal-Ottawa scale		
		Types of gastric cancer in ICG group	Types of gastric cancer in non-ICG group	Types of operation in ICG group	Types of operation in non-ICG group	Number, mean and standard deviation of participants in ICG group			Number, mean and standard deviation of participants in non-ICG group				Selection	Comparability	Exposure
						Number of participants in ICG group	Mean number of lymph node dissected in ICG group	Standard deviation of number of lymph node dissected in ICG group	Number of participants in non-ICG group	Mean number of lymph node dissected in non-ICG group	Standard deviation of number of lymph node dissected in non-ICG group				
**2020-Shin-Hoo Park et al.** (27)	Korea	Adenocarcinoma	Adenocarcinoma	ICG guided conventional laparoscopic gastrectomy and D2 lymph node dissection	Conventional laparoscopic gastrectomy and D2 lymph node dissection without ICG tracer	20	30.15	9.27	60	32.55	10.03	Prospective cohort study	★★★☆	★★	★☆☆
**2020-Chen et al.** (24)	China	Adenocarcinoma	Adenocarcinoma	ICG guided conventional laparoscopic gastrectomy and D2 lymph node dissection	Conventional laparoscopic gastrectomy and D2 lymph node dissection without ICG tracer	129	50.5	15.9	129	42	10.3	prospective cohort study	★★★★	★★	★★☆
**2018-InGyu Kwon et al**. (21)	American	Adenocarcinoma	Adenocarcinoma	ICG guided da Vinci Si Surgical System (robotic) laparoscopic gastrectomy and D2 lymph node dissection	da Vinci Si Surgical System (robotic) laparoscopic gastrectomy and D2 lymph node dissection without ICG tracer	40	48.9	14.6	40	35.2	11.2	Prospective cohort study	★★★☆	★★	★☆☆
**2019-Fabio Cianchi et al.** (32)	Italy	Intestinal, diffuse, or mixed gastric cancer	Intestinal, diffuse, or mixed gastric cancer	ICG guided da Vinci Si Surgical System (robotic) laparoscopic gastrectomy and D2 lymph node dissection	da Vinci Si Surgical System (robotic) laparoscopic gastrectomy and D2 lymph node dissection without ICG tracer	37	4	5.4	37	4.4	6.8	prospective cohort study	★★★★	★★	★☆☆
**2019- Ma et al.** (28)	China	Adenocarcinoma	Adenocarcinoma	ICG guided conventional laparoscopic gastrectomy and D2 lymph node dissection	Conventional laparoscopic gastrectomy and D2 lymph node dissection without ICG tracer	38	2	10	44	2.5	14.75	retrospective cohort study	★★★☆	★☆	★☆☆
**2020-Ma et al.** (35)	China	Primary advanced gastric cancer	Primary advanced gastric cancer	ICG guided conventional laparoscopic gastrectomy and D2 lymph node dissection	Conventional laparoscopic gastrectomy and D2 lymph node dissection without ICG tracer	31	9	11.2	34	6.8	6.9	retrospective cohort study	★★★☆	★☆	★☆☆
**2018-Yuki Ushimaru et al.** (36)	Japan	Adenocarcinoma	Adenocarcinoma	ICG guided conventional laparoscopic gastrectomy and D2 lymph node dissection	Conventional laparoscopic gastrectomy and D2 lymph node dissection without ICG tracer	84	47.5	1.7	84	42.6	1.7	retrospective cohort study	★★★☆	★★	★☆☆
**2019-Tu et al.** (37)	China	Adenocarcinoma or signet ring cell carcinoma	Adenocarcinoma or signet ring cell carcinoma	ICG guided conventional laparoscopic gastrectomy and D2 lymph node dissection	Conventional laparoscopic gastrectomy and D2 lymph node dissection without ICG tracer	39	2	10.5	663	1	17	retrospective cohort study	★★★☆	★☆	★★★
**2020-Liu et al.** (38)	China	Primary gastric cancer	Primary gastric cancer	ICG guided conventional laparoscopic gastrectomy and D2 lymph node dissection	Conventional laparoscopic gastrectomy and D2 lymph node dissection without ICG tracer	61	1.56	3.21	75	1.44	2.66	retrospective cohort study	★★★☆	★☆	★☆☆
**2017-Yuan-Tzu Lan et al.** (29)	China	Adenocarcinoma	Adenocarcinoma	ICG guided da Vinci Si Surgical System (robotic) laparoscopic gastrectomy and D2 lymph node dissection	da Vinci Si Surgical System (robotic) laparoscopic gastrectomy and D2 lymph node dissection without ICG tracer	14	35.8	11.4	65	30.0	11.8	retrospective cohort study	★★★★	★☆	★☆☆
**2021-Zening Huang et al.** (25)	China	Adenocarcinoma	Adenocarcinoma	ICG guided conventional laparoscopic gastrectomy and D2 lymph node dissection	Conventional laparoscopic gastrectomy and D2 lymph node dissection without ICG tracer	94	40.8	13.7	94	31.8	13.5	retrospective cohort study	**★★★★**	**★★**	**★★☆**
**2020-Yuan Tian et al.** (34)	China	Intestinal, diffuse, or mixed gastric cancer	Intestinal, diffuse, or mixed gastric cancer	ICG guided da Vinci Si Surgical System (robotic) laparoscopic gastrectomy and D2 lymph node dissection	da Vinci Si Surgical System (robotic) laparoscopic gastrectomy and D2 lymph node dissection without ICG tracer	27	39.19	8.97	32	35.28	9	retrospective cohort study	**★★★★**	**★★**	**★★☆**
**2020-Xiaofeng Lu et al.** (33)	China	Adenocarcinoma	Adenocarcinoma	ICG guided conventional laparoscopic gastrectomy and D2 lymph node dissection	Conventional laparoscopic gastrectomy and D2 lymph node dissection without ICG tracer	28	27.5	10.6	28	21.79	6.73	retrospective cohort study	**★★★★**	**★★**	**★★☆**

★, score point.

☆, no score point.

### 2.1 Information sources

A systematic review was conducted in PubMed, Embase, Web of Science, the Cochrane Library, and Scopus to identify relevant subjects from inception to June 2022. The references for relevant systematic reviews and meta-analysis are also cited.

### 2.2 Search strategy

The search strategy, including a medical subject heading (MeSH) and its free terms, was as follows:

- Stomach Neoplasma: “stomach neoplasm” or “gastric cancer” OR “gastric neoplasm” or “stomach cancer” or “cancer of the stomach” or “cancer of stomach.”
- Indocyanine Green: “indocyanine green” or “wofaverdin” or “vophaverdin” or “ujoveridin” or “vofaverdin” or “Cardio-Green” or “Cardio Green” or “Cardiogreen.”
- Finally, i. and ii. are connected by the operator “AND.”

### 2.3 Selection criteria

We retrieved studies that compared the efficacy and/or safety of ICG in laparoscopic gastrectomy with that of conventional laparoscopic gastrectomy. All retrieved studies were loaded into the reference management software NoteExpress 3.2.0.7276. There were no language limitations in the selection criteria. Duplicate studies were checked and removed. The remaining studies were checked through a preliminary screening based on the relevance of the title, abstract, and keywords. The remaining studies after preliminary screening were checked again based on the inclusion and exclusion criteria. Subsequently, the full text was checked carefully and deliberately. The Problem/patient, Intervention, Comparison, Outcome, Study design (PICOS) method (30) was used for inclusion and exclusion criteria.

The inclusion criteria are as follows:

- The tumor type of the patient was gastric cancer.
- Indocyanine green was used as a tracer in laparoscopic gastrectomy in the interventional group;
- The control group underwent conventional laparoscopic gastrectomy;
- Studies provide exact statistical data such as the mean number and standard deviation of lymph nodes, or enough data for these measures to be calculated.
- Studies have reported the number and region of lymph nodes dissected.

The exclusion criteria are as follows:

- Patients with early gastric carcinoma
- Patients who had a history of gastric surgery
- The patients were younger than 18
- Studies are not original articles (reviews, case reports, comments, and so on)
- Studies of low quality.

### 2.4 Data collection process

The assessment of the efficacy of laparoscopic gastrectomy was mainly based on the number of lymph nodes dissected. In our study, we collected the number of patients, the mean number of lymph nodes dissected, and the standard deviation of the mean number of lymph nodes dissected. Then the pooled effect sizes were calculated using the weighted mean difference (WMD) and 95% confidence interval (CI) according to related algorithm data (31).

Based on the data below, WMDs were obtained. WMDs were used to assess the difference in efficacy of different interventions.

JZ and KL designed the search strategy. CS and YZ checked and modified the search strategy. JZ and KL retrieved literature based on the search strategy. ZJ, KL, ZW, QK, LJ, YZ, and XZ preliminarily screened the retrieved literature. ZW and QK searched for the full text. JZ and KL made the final decision to include literature. Agreements were reached by consensus through discussion with experienced researchers (CS and YZ).

### 2.5 Data items

The following data were extracted: year of publication; the first author; the country or area of study; the number of participants; male/female; mean age of participants; type of gastric cancer; the region of lymph node dissected; group of lymph node; types of laparoscopic of control group; the method of lymph node dissection; injection timing of indocyanine green; injection site of indocyanine green; injection dosage of indocyanine green; usage of indocyanine green; optical imaging mode; mean, standard deviation and interval value of lymph node number; the source of participants; drug dose of ICG; mean tumor diameter; time of operation, intraoperative blood loss, and postoperative hospitalization.

### 2.6 Risk of bias in individual studies

Group A (JZ and ZW) and Group B (KL and QK) both used the Newcastal-Ottawa scale (NOS) to evaluate the quality of included studies. Part of the assessment of case–control studies consists of selection, comparability, and exposure. The part of assessment of cohort study consists of selection, comparability and outcome. Any disagreements were resolved by discussion with two experts (YZ and CS).

### 2.7 Summary measures

The Weighted Mean Difference (WMD) and confidential interval (95% CI) were outcome indicators in the present systematic review and meta-analysis.

### 2.8 Synthesis of results

The pooled WMD was calculated for laparoscopic gastrectomy with ICG and without ICG in each study. The pooled WMD presents with 95% CIs. Subgroup analysis was performed to provide more explicit results. We measured heterogeneity among the included studies by the *I* ^2^ test and χ^2^ statistic. In the χ^2^ statistic, a *P*-value <0.1 was considered significant. A random-effects model was used to combine the effect sizes of the included studies if significant heterogeneity (*P <*0.1 or *I* ^2^ >50%) existed. Meanwhile, significant heterogeneity was analyzed by stratified analysis, sensitivity analysis, and meta-regression. Sensitivity analysis, stratified analysis, and meta-regression were performed to evaluate the robustness of the included papers. Publication bias was evaluated using the Egger test and funnel plot. Statistical analyses were performed by STATA version 12.0 (StataCorp LP, College Station, TX, USA).

### 2.9 Risk of bias across studies

We determined the presence of publication bias using the Egger’s test and Begg’s test, or funnel plots if necessary.

### 2.10 Additional analyses

Stratified analysis was performed where appropriate to analyze the source of heterogeneity.

## 3 Results

### 3.1 Selection of study

**Figure 1:**
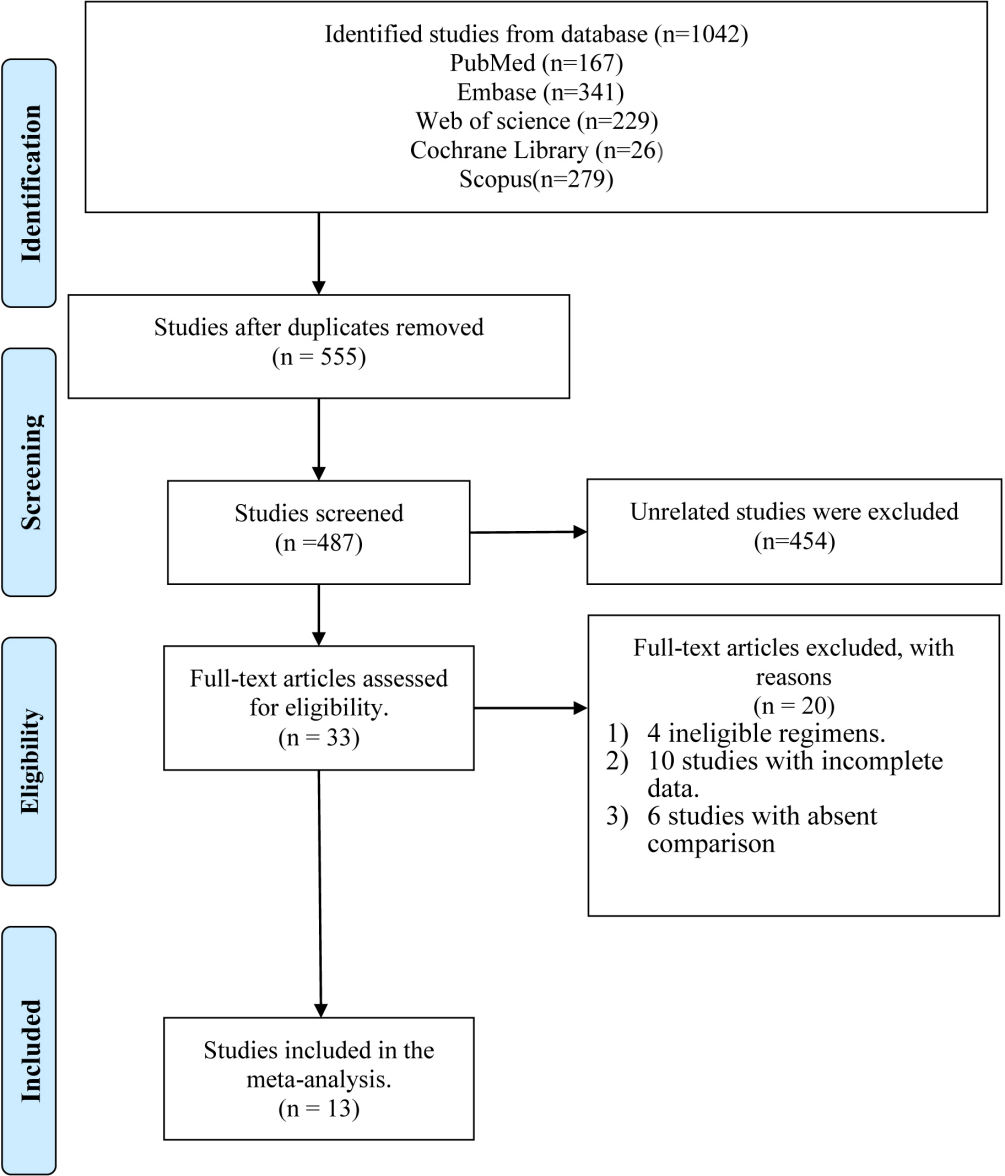
Results of literature retrieval.

The strategic search yielded a total of 1,042 references, among which 167 references were retrieved and identified from PubMed, 341 from Embase, 229 from Web of Science, 26 from the Cochrane library, and 279 from Scopus. Of these articles, 555 duplicate references were excluded at first. We further excluded another 454 unrelated references after preliminary screening based on the corresponding title, abstract, and keywords. Then 33 relevant articles were selected and reviewed by two independent authors (ZJ and LK). After reviewing the full text of articles, 20 references were excluded: two with ineligible regimens, ten references with incomplete data, and six without comparable groups. Finally, 13 articles (21, 24, 25, 27–29, 32–38) were found to meet the inclusion criteria and were thus included for further analysis. The process of study selection is explained in a flow diagram (**Figure 1**).

### 3.2 Study characteristics and risk of bias within studies

Detailed characteristics of the included articles are shown in **Table 1**. Thirteen articles included 2,027 participants (642 for the ICG group and 1,385 for the non-ICG group). Nine studies (24, 25, 27, 28, 33, 35–38) compared the efficacy and safety of ICG tracer-guided lymph node dissection in laparoscopic radical gastrectomy versus conventional laparoscopic gastrectomy for gastric cancer with the help of a robot, and the other four studies (21, 29, 32, 34) compared it with robotic laparoscopic gastrectomy without the help of a robot (**Table 1**). For the type of research design, all (13/13) of them were cohort studies (analytical research) (**Table 1**). Nine studies (24, 25, 28, 29, 33–35, 37, 38) were conducted in China, while the rest were conducted in Korea (27), Japan (36), America (21), and Italy (32), respectively (**Table 1**). All the patients were diagnosed with gastric cancer. In terms of patient age, it ranged from 50 to 80 (**Table S1**). In terms of tumor site, there were longitudinal and circumferential resection margins to classify. Three studies (27, 29, 36) were classified through both longitudinal and circumferential resection margins (**Table S7**). Eight (21, 24, 25, 32–34, 37, 38) studies were classified through longitudinal resection margins (**Table S7**). Two studies (28, 35) lacked tumor site data. In terms of pathological type, ten studies (21, 24, 25, 27–29, 33, 34, 36, 37) were adenocarcinoma (**Table S6**). Three studies (32, 35, 38) lacked data of the pathological type. In terms of diagnostic criteria of tumor size, seven studies (21, 25, 27, 29, 32, 33, 38) were diagnosed by the seventh edition of the classification guidelines issued by the American Joint Committee on Cancer (39) (**Table S6**). Three studies (24, 28, 37) were diagnosed by the eighth edition of the classification guidelines issued by the American Joint Committee on Cancer (40) (**Table S6**). Two studies (34, 36) were diagnosed with Japanese Gastric Cancer A. Japanese classification of gastric carcinoma: 3rd English edition (41) (**Table S6**). One study (35) lacked the data for diagnostic criteria of tumor size. In terms of the method of optical imaging of indocyanine green, eleven studies (21, 24, 25, 27, 29, 32–37) were near-infrared (NIR) imaging systems (**Table S1**). Two studies (28, 38) were fluorescence surgical systems (**Table S1**). In terms of injection site of Indocyanine green, eleven studies (21, 24, 27, 28, 32–38) were injected indocyanine in submucosal (**Table S5**). One study (25) injected indocyanine into the subserosa (**Table S5**). Another study (29) injected indocyanine into both the submucosal and subserosa (**Table S5**). In terms of injection timing of indocyanine green, one study (27) injected intraoperatively (**Table S5**). Eleven studies (21, 24, 25, 28, 32–38) were injected preoperatively. One study (27) was injected intraoperatively. One study (29) included intraoperative and preoperative injections (**Table S5**). The methods of gastrectomy included radical total gastrectomy, radical subtotal gastrectomy, proximal gastrectomy, and distal gastrectomy (**Table S6**). Five kinds of concentration of ICG were used in the included studies (**Table S5**). One (36) was 0.05 g/L (**Table S5**). One (27) was 0.1 g/L (**Table S5**). One (25) was 0.5 g/L (**Table S5**). One (38) was 0.625 g/L (**Table S5**). Six (21, 24, 28, 32, 35, 37) were 1.25 g/L (**Table S5**). Three (29, 33, 34) are 2.5 g/L (**Table S5**). The mean number of lymph nodes dissected and its standard deviation were used as the endpoint of studies in all included studies. The efficacy indications, which include the number of patients, the mean number of lymph nodes dissected, and the standard deviation of the mean number of lymph nodes dissected, are listed in **Table 1**. The safety indications, including time of operation, intraoperative blood loss, and postoperative hospitalization, are listed in **Tables S2**-**S4**. There was some missing information. Two studies did not provide the body mass index of participants, the sex ratio of participants, or the mean diameter of the tumor. Information about the risk of bias within studies is shown in **Table 1**.

### 3.3 Efficacy of indocyanine green in laparoscopic gastrectomy of gastric cancer

#### 3.3.1 Laparoscopic gastrectomy of gastric cancer with indocyanine green

**Figure 2:**
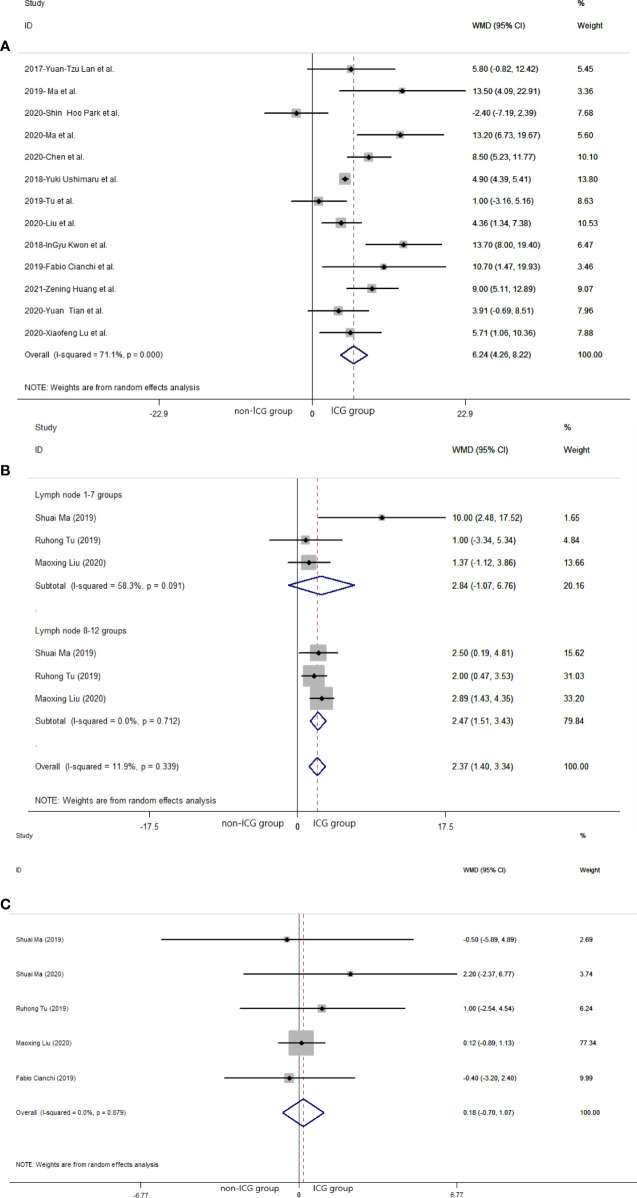
Pooled weighted mean difference of efficacy of included studies related to laparoscopic gastrectomy with indocyanine green *vs*. conventional laparoscopic gastrectomy. **(A)** Pooled weighted mean difference of efficacy of included studies related to indocyanine green. **(B)** Pooled weighted mean difference of efficacy of included studies related to indocyanine green in different lymph node groups. **(C)** Pooled weighted mean difference of efficacy of included studies related to positive (metastatic) lymph node.

Regarding the 13 studies (21, 24, 25, 27–29, 32–38) that analyzed the efficacy of laparoscopic gastrectomy with ICG, the mean number of lymph nodes dissected in the ICG group was significantly greater than that in the non-ICG group (WMD = 6.24, 95% CI: 4.26 to 8.22) (**Figure 2A**). The heterogeneity was statistically significant (*I* ^2^: 71.1% and *P*-value: <0.001) (**Figure 2A**). There was no significant difference between the mean number of dissected lymph nodes in the laparoscopic gastrectomy of lymph node 1–7 groups in the ICG group and that in the non-ICG group (WMD = 2.84, 95% CI: −1.07 to 6.76) (**Figure 2B**). The mean number of dissected lymph nodes in the laparoscopic gastrectomy of lymph node 8 to 12 groups with ICG was greater than that in the non-ICG group (WMD = 2.47, 95% CI: 1.51 to 3.43) (**Figure 2B**). There was no significant difference between the mean number of positive (metastatic) lymph nodes dissected in the ICG group and that in the non-ICG group (WMD = 0.18, 95% CI: −0.70 to 1.07) (**Figure 2C**).

##### 3.3.1.1 Subgroup analysis: Year of publication (2017–2018, 2019–2021)

**Figure 3:**
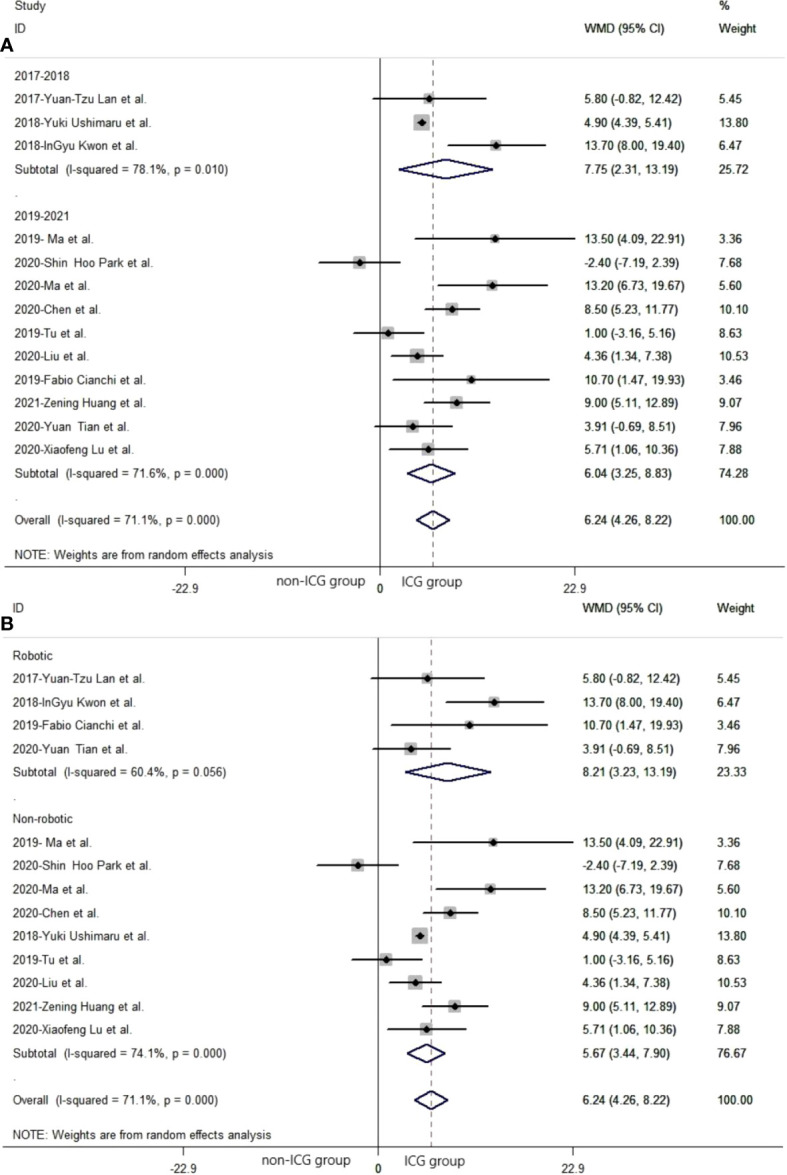
The pooled weighted mean difference of efficacy of included studies related to subgroup analysis. **(A)** The pooled weighted mean difference of efficacy of included studies related to different year of publication. **(B)** The pooled weighted mean difference of efficacy of included studies related to different types of laparoscopic gastrectomy.

In subgroup analysis related to different publication years, 2017 to 2018 and 2019 to 2021, the pooled number of lymph nodes in laparoscopic gastrectomy with ICG between 2017 and 2018 was significantly more than that in the non-ICG group (WMD = 7.75, 95% CI: 2.31 to 13.19) (**Figure 3A**). The pooled number of lymph nodes in laparoscopic gastrectomy with ICG between 2019 and 2021 was significantly greater than that in the non-ICG group (WMD = 6.04, 95% CI: 3.25 to 8.83) (**Figure 3A**).

##### 3.3.1.2 Subgroup analysis: Robotic gastrectomy and non-robotic gastrectomy

In subgroup analysis related to different laparoscopic gastrectomy techniques (robotic and non-robotic), the mean number of dissected lymph nodes in robotic laparoscopic gastrectomy with ICG was significantly greater than that in the non-ICG group (WMD = 8.21, 95% CI: 3.23 to 13.19) (**Figure 3B**). The mean number of dissected lymph nodes in conventional laparoscopic gastrectomy with ICG was significantly greater than that in the non-ICG group (WMD = 5.67, 95% CI: 3.44 to 7.90) (**Figure 3B**).

### 3.4 Safety of laparoscopic gastrectomy with indocyanine green

**Figure 4:**
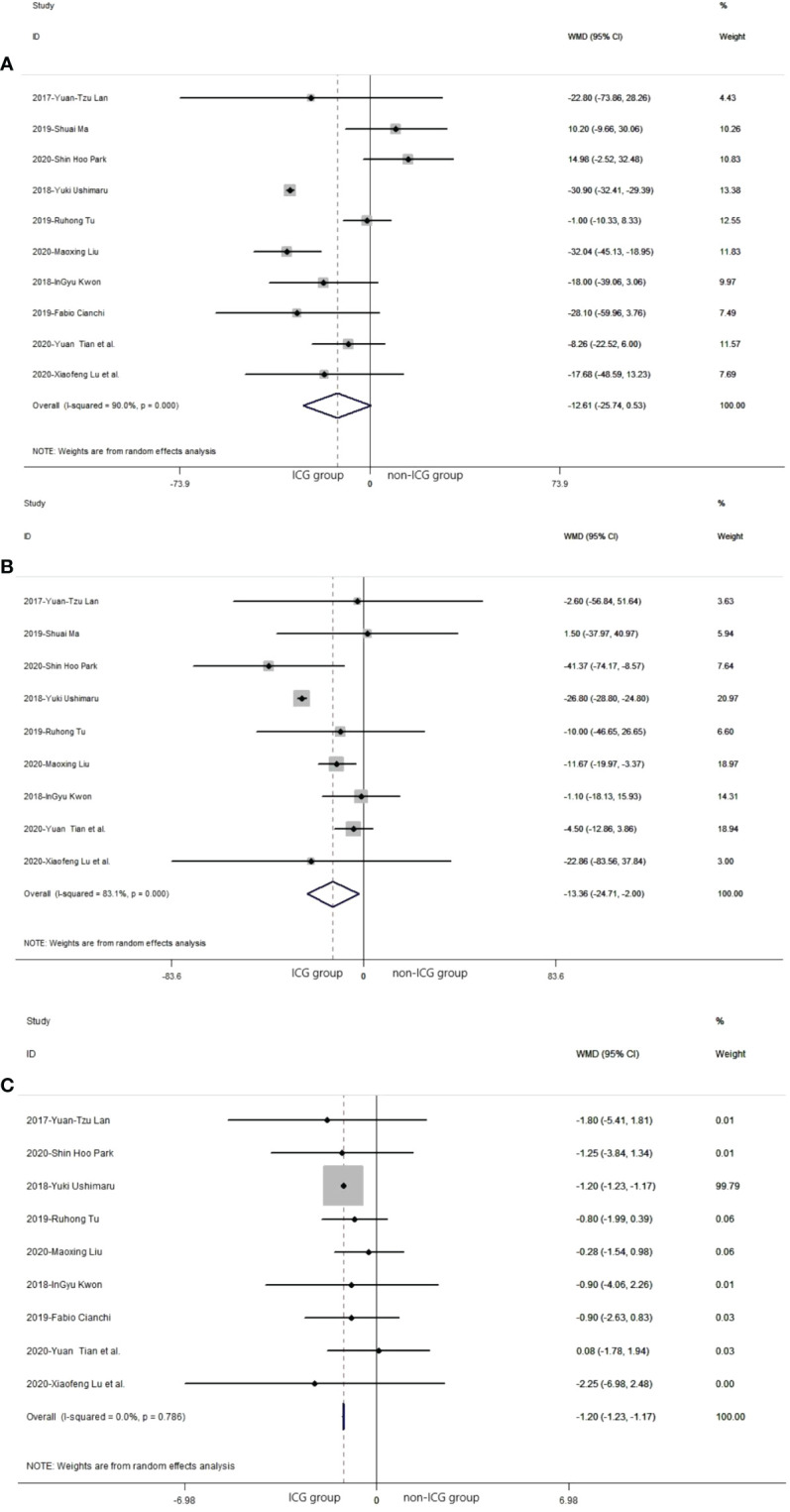
Pooled weighted mean difference of safety in laparoscopic gastrectomy with indocyanine green *vs*. conventional laparoscopic gastrectomy. **(A)** The pooled weighted mean difference of safety of included studies related to time of operation. **(B)** The pooled weighted mean difference of safety of included studies related to intraoperative blood loss. **(C)** The pooled weighted mean difference of safety of included studies related to time of postoperative hospitalization.

In the 10 studies that analyzed the safety of the laparoscopic gastrectomy with ICG, the mean time of operation in the laparoscopic gastrectomy with ICG was shorter than that in the non-ICG group (WMD = −12.61, 95% CI: −25.74 min to 0.53 min) (**Figure 4A**). The mean volume of intraoperative blood loss in laparoscopic gastrectomy with ICG was significantly less than that in the non-ICG group (WMD = −13.36, 95% CI: −24.71 to −2.00) (**Figure 4B**). The mean time of postoperative hospitalization in laparoscopic gastrectomy with ICG was significantly shorter than that in the non-ICG group (WMD = −1.20, 95% CI: −1.23 to −1.17) (**Figure 4C**).

### 3.5 Meta-regression, stratified analysis, and sensitivity analysis

**Figure 5:**
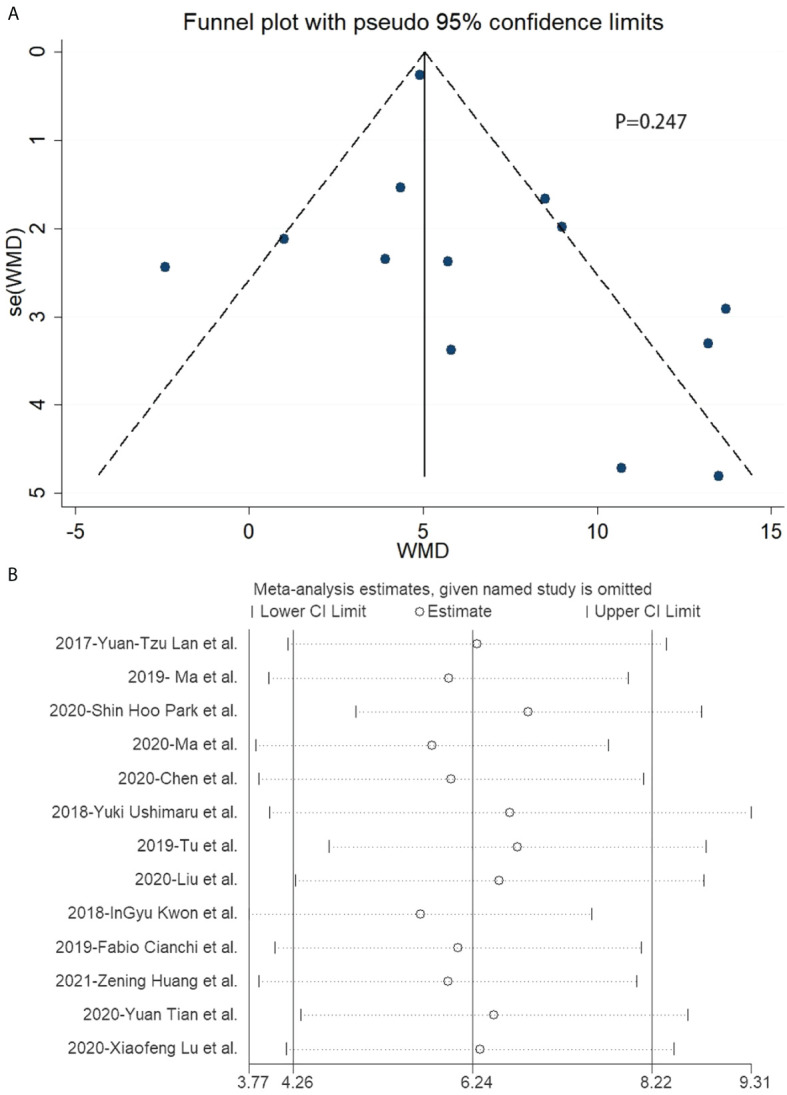
Funnel plot and sensitivity analysis of included studies related to laparoscopic gastrectomy with indocyanine green. **(A)** Funnel plot of included studies related to laparoscopic gastrectomy with indocyanine green. **(B)** Sensitivity analysis of included studies related to laparoscopic gastrectomy with indocyanine green.

The study performed meta-regression and stratified analysis for types of laparoscopic gastrectomy and year of publication. The types of laparoscopic gastrectomy were divided into robotic laparoscopic gastrectomy and conventional laparoscopic gastrectomy (**Table 2**). In the meta-regression of types of laparoscopic gastrectomy, the p-value was 0.455 (>0.05). For the heterogeneity of different subgroups of types of laparoscopic gastrectomy, there was no statistical significance (*P* >0.05). The year of publication was segmented into 2017–2018 and 2019–2021 (**Table 2**). In the meta-regression of the year of publication, the p-value is 0.618. In the heterogeneity of different subgroups of the year of publication, there was no statistical significance (*P* >0.05). In sensitivity analysis, there is no meaningful change in removing each study in turn and recalculating the pooled estimate for all remaining studies (**Table 2**) (**Figure 5B**).

**Table 2:** Meta-regression and stratified analysis of included studies related to laparoscopic gastrectomy with indocyanine green.

	Stratified			Meta-regression			
	No. of studies	Pooled WMD(95% CI)	Heterogeneity(p-Value)		Coefficient		p-Value	
**Crude**	13	6.240 (4.258, 8.221)	<0.001					
**Laparoscopy types**					−2.354495		0.455	
**robotic**	4	8.213 (3.231, 13.194)	0.056					
**non-robotic**	9	5.208 (2.528, 7.887)	<0.001					
**Year of** **publication**					−1.620863		0.618	
**2017-2018**	3	7.747 (2.307, 13.186)	0.010					
**2019-2020**	10	6.038 (3.246, 8.830)	<0.001	

ICG, indocyanine green; WMD, weighted mean difference; CI, confidence interval; NIR, near-infrared light; PRISMA, Preferred Reporting Items for Systematic Reviews and Meta-Analyses; PICOS; Problem/patient, Intervention, Comparison, Outcome, Study design; Y, yes; N, no; U, unclear.

### 3.6 Risk of publication bias across studies

In the included studies related to laparoscopic gastrectomy with ICG, there was no publication bias in the funnel plot (**Figure 5A**). Furthermore, Egger’s test revealed no significant publication bias (*P* = 0.247) (**Figure 5A**).

## 4 Discussion

A systematic review and meta-analysis were conducted to determine the safety and efficacy of ICG in laparoscopic gastrectomy. Pooled results of different studies showed that, in terms of efficacy, the ICG tracer strengthened lymph node clearance in laparoscopic gastrectomy. It has the advantage of allowing surgeons to see and dissect lymph nodes more easily, which reduces the risk of metastasis and impedes gastric cancer progression. ICG can help to identify more lymph nodes than the naked eye, thus helping to guide lymph node dissection. However, according to the mechanism of ICG, it is undeniable that ICG can only identify lymph nodes, but whether the lymph nodes are malignant (positive) or not must be removed empirically and finally determined by pathology. This lymph node resection during cancer surgery is generally performed for two main reasons: a) staging and b) dissemination prevention. Thus, the number of resected nodes increases with the number of suspicious nodes (up to a certain limit) and with the striving for dissemination prevention. In the first case, more nodes might indicate a bad prognosis, while in the latter, better dissemination prevention might be achieved by exciding more nodes. However, the number of examined lymph nodes remains controversial in predicting survival. This problem deserves further discussion in the follow-up research. In terms of safety, the ICG tracer reduces intraoperative blood loss and postoperative hospitalization. Additionally, the ICG tracer could reduce the time of operation because only an extremely small proportion of 95% CI includes 0. The results below indicate ICG could be a good tracer in laparoscopic gastrectomy.

The study assessed the efficacy of laparoscopic gastrectomy based on the number of lymph nodes dissected. Our results indicate ICG tracer increases the mean number of lymph nodes dissected, particularly in robotic laparoscopic gastrectomy. Through subgroup analysis, no statistical difference was found in station 1 (1–7 group) lymph node dissection between different interventions. However, in station 2 (8–12 groups) lymph node dissection, there is a statistical difference between different interventions. The extent of D1 lymph node dissection was lymph node 1–7 groups (42). Lymph node 8–12 groups is the extent of lymph node dissection in D2 lymph node dissection (42). It indicates ICG tracer is more suitable for D2 lymph node dissection than D1 lymph node dissection, which was beneficial to improving the living conditions and prolonging the survival time of patients. Some studies found D2 lymph node dissection had some advantages, including low morbidity and high survival rates (43, 44). A trend of improved survival exists among D2 patients who did not undergo resection of the spleen or pancreas, as well as for patients with T3/T4 cancers. However, the survival time has not been investigated as an outcome of the present meta-analysis due to a lack of the follow-up data reported. Therefore, further research is warranted to explore the potential long-term survival benefit of ICG tracer-guided lymph node dissection in minimally invasive radical gastrectomy. In recent years, D2 lymph node dissection has been widely accepted as a standard for advanced or early resectable gastric cancer with lymph node metastasis, especially in Asia. Meanwhile, ICG studies were mostly reported from Asia. The lymph nodes are mainly distributed along the blood vessels that determine the importance of dealing with blood vessels for laparoscopic radical gastric cancer surgery, so the precise positioning of lymph nodes is critical in laparoscopic radical gastrectomy. ICG may improve the identification of lymph nodes in D2 gastrectomy. At present, there is no consensus on how to effectively operate D2 lymph node dissection in the world. Based on the research data, ICG was suggested to be used as a tracer material for D2 lymph node dissection. There was no statistical difference in the number of positive (metastatic) lymph nodes dissected between different interventions. ICG cannot just recognize more positive lymph nodes but also recognize more lymph nodes. To a certain extent, the more lymph nodes identified, the better the positive rate of dissection. In addition, effective dissection of lymph nodes means smooth operation and fewer complications due to the occultation of lymph nodes and their close relationship with blood vessels. Hence, we still thought laparoscopic gastrectomy with ICG had an edge over conventional laparoscopic gastrectomy in lymph node dissection and reduced complications. Of course, tracers with better affinity or targeting to positive lymph nodes may be available in the near future with the improvement of biomaterials. We hope to find better tracers for positive (metastatic) lymph node dissection in the future.

This study assessed the safety of laparoscopic gastrectomy based on time of operation, intraoperative blood loss, and postoperative hospitalization. There was a significant statistical difference in intraoperative blood loss and postoperative hospitalization. Although there was no statistical difference in the time of operation, only an extremely small proportion of 95% CI included 0. ICG could be acceptably used in laparoscopic gastrectomy.

There was no significant publication bias in the included data according to Egger’s test, while the funnel plot showed publication bias. Publication bias indicted by the funnel plot may be caused by the small sample size of included studies, which means more high-quality studies were needed in this research. There was significant heterogeneity in the included data. Hence, the study performed a stratified analysis and metaregression of two factors: surgical operation mode and different publication years. Surgical operations were classified as robotic and non-robotic. The publication years were divided into 2017–2018 and 2019–2020. However, no unambiguous results found the source of heterogeneity.

Gastric cancer is a great burden on society, so it is necessary to improve the treatment of gastric cancer. Advanced treatment technology can improve the survival rate of cancer patients and reduce the mortality rate (45). For undifferentiated adenocarcinomas and submucosal tumors, standard gastrectomy and lymphadenectomy should be performed as far as possible (46). ICG has been used as a new tracer agent in many malignant tumor surgeries (8). It fluoresced after the stimulus using a laser beam of 820 nm, or near-infrared light (NIR). Infrared light has a longer wavelength and can penetrate the thick fatty tissues of the body better, so it can see the lymph nodes better than other methods (21). It has been shown in the literature that standard lymph node dissection can improve survival in patients with gastric cancer (47). It is encouraging to see that more and more new techniques are being put into the clinical treatment of gastric cancer.

Compared with previous meta-analyses, we analyzed the dissection results of all lymph nodes, not just sentinel lymph node dissection. We also collected the data from different years and compared the number of dissected lymph nodes. We also compared the data from robotic surgery to non-robotic surgery. But there are certain limitations to our systematic review and meta-analysis. Most of the included literature only reported comparisons between ICG imaging and conventional surgery, while there were few reports on lymph node dissection using alternative staining methods in the non-ICG group. Meanwhile, there are few studies of the dissection of lymph node 8–12 groups to compare laparoscopic gastrectomy using ICG and conventional laparoscopic gastrectomy. More studies are needed to prove the efficacy of dissection of lymph node 8–12 groups in laparoscopic gastrectomy using ICG.

The application of ICG in laparoscopic/robotic lymphadenectomy for GC patients is still in the preliminary stage, and most of the published studies on this issue have a limited sample size. So far, only one study was a randomized controlled trial (RCT). Although the level of the evidence is relatively low, it does not affect the value of the results of this systematic review and meta-analysis. In particular, whether the use of ICG-guided minimally invasive lymphadenectomy can improve the total number of harvested lymph nodes is still unclear.

For the security and effectiveness of ICG, we need a lot of comparisons to verify its effect. In future studies, multiple comparisons should be studied as much as possible, and the surgical methods should be unified. Secondly, most of the included literature was from eastern countries. This is an obvious limitation of our study. These types of studies are generally not done in western countries. Therefore, our findings may not apply to the entire global population. Relevant research should also be carried out in the West to obtain more meaningful results. We believe that the difference in surgical methods may affect the number of dissected lymph nodes. But due to the data deficiencies, we could not conduct efficacy-stratified analysis. Here, if the data prove the effectiveness of ICG in lymph node dissection for gastric cancer, it may be of great help to the future treatment of gastric cancer.

## 5 Conclusion

Existing evidence suggests that the ICG tracer increases the number of lymph node dissections during laparoscopic radical gastrectomy with D2 lymphadenectomy for gastric cancer. Moreover, the use of ICG not only has no undesirable implications but also contributes to the safety of conventional laparoscopic gastrectomy. Therefore, ICG-assisted laparoscopic lymph node dissection is a promising option. Nevertheless, more prospective studies and long-term follow-up are still necessary to address the limitations of this evidence.

## Data availability statement

The original contributions presented in the study are included in the article/**Supplementary Material**. Further inquiries can be directed to the corresponding authors.

## Author contributions

CS and YZ conceived and designed the research. JZ and KL analyzed the data and wrote the manuscript. ZW, QK, JL, YZ, and XZ performed the research. CS and YZ edited the manuscript. All authors listed have made a substantial, direct, and intellectual contribution to the work and approved it for publication.

## Funding

Funding was provided by the National Natural Science Foundation of China (grant number 81860099), the Natural Science Foundation of Fujian Province of China (grant number 2017J01347), and the Science Foundation of Putian University of Fujian Province China (grant number 2016049).

## Conflict of interest

The authors declare that the research was conducted in the absence of any commercial or financial relationships that could be construed as a potential conflict of interest.

## Publisher’s note

All claims expressed in this article are solely those of the authors and do not necessarily represent those of their affiliated organizations, or those of the publisher, the editors and the reviewers. Any product that may be evaluated in this article, or claim that may be made by its manufacturer, is not guaranteed or endorsed by the publisher.
