# Supplementary material for: Efficacy and safety of indocyanine green tracer-guided lymph node dissection in minimally invasive radical gastrectomy for gastric cancer: A systematic review and meta-analysis
Source: Front Oncol. 2022 Aug 5;12:884011. doi: 10.3389/fonc.2022.884011 (PMC9388933; doi:10.3389/fonc.2022.884011)
Supplement: Supplementary file 1 [file Table_1.docx]

**Table S1** Characteristics of included studies.

| Year-Author | Country | Region of lymph node dissected | The method of optical imaging | Male/Female (ICG group/non-ICG group) | mean age (years old) (ICG group/non-ICG group) | Source of participants | Mean tumor diameter(mm) (ICG group/non-ICG group) | BMI of ICG group(kg/m^2^) | BMI of non-ICG group(kg/m^2^) |
| --- | --- | --- | --- | --- | --- | --- | --- | --- | --- |
| 2017-Yuan-Tzu Lan et al. | China | Group 1-12 | Near-infrared (NIR) imaging system | (7/7)/ (38/27) | (66.0±12.4)/ (67.8±15.6) | The Institutional Review Board of Taipei Veterans General Hospital | (37±17)/(34±16) | 24.0±4.1 | 24.4±3.1 |
| 2019- Ma et al. | China | Group 1-12 | Storz fluorescent laparoscopic | (29/9)/ (31/13) | (59.7±9.1)/ (57.3±12.0) | Department of Pancreatic and Gastric Surgery, Cancer Hospital, Chinese Academy of Medical Sciences | - | 23.7±2.9 | 23.1±3.1 |
| 2020-Shin‑Hoo Park et al. | Korea | Group 1-12 | Near-infrared (NIR) imaging system | (15/5)/ (45/15) | (60.10±11.09)/ (61.67±11.47) | Seoul National University Hospital | (30.3±12.5)/ (28.2±16.0) | 24.89±3.22 | 24.09±2.80 |
| 2020-Ma et al. | China | Group 1-12 | Near-infrared (NIR) imaging system | - | - | National Cancer Center, China | - | - | - |
| 2020-Chen et al. | China | Group 1-12 | Near-infrared (NIR) imaging system | (86/43)/ (87/42) | (57.8±10.7)/ (60.1±9.1) | Fujian Medical University Union Hospital | (65 participants with tumor diameter greater than 30mm and 64 participants with that less than 30mm.)/ (79 participants with tumor diameter greater than 30mm and 50 participants with that less than 30mm.) | 23.2±3.2 | 22.8±3.1 |
| 2018-Yuki Ushimaru et al. | Japan | Group 1-12 | Near-infrared (NIR) imaging system | (47/37)/ (46/38) | (66.2±1.2)/ (66.6±1.2) | Department of Surgery, Osaka International Cancer Institute, Osaka, Japan | (42.6±2.3)/ (38.7±2.3) | 22.92±0.35 | 22.83±0.35 |
| 2019-Tu et al. | China | Group 1-12 | Near-infrared (NIR) imaging system | (26/13)/ (491/172) | (58±13)/ (61±11) | Fujian Medical University Union Hospital | (40.0±23)/ (43.0±33) | 23±4 | 24±4 |
| 2020-Liu et al. | China | Group 1-12 | NOVADAQ fluorescence surgical system | (33/28)/ (47/28) | (55.11±10.76)/ (58.40±10.71) | Peking University Cancer Hospital & Institute, Beijing, China. | (26)/ (26.8) | 23.75±3.49 | 23.51±2.51 |
| 2018-InGyu Kwon et al. | American | Group 1-12 | Near-infrared (NIR) imaging system | (21/19)/ (19/21) | (52.2±11.7)/ (52.1±11.3) | The Institutional Review Board of Severance Hospital, Yonsei University College of Medicine, Seoul, Korea | - | - | - |
| 2019-Fabio Cianchi et al. | Italy | Group 1-12 | Near-infrared (NIR) imaging system | (22/15)/ (21/16) | (72.2±9.8)/ (72.4±8.9) | The Center for Oncologic Minimally Invasive Surgery of the University of Florence, Italy. | (38±19)/ (39±21.9) | 23.3±3.07 | 23.2±3.04 |
| 2021-Zening Huang et al. | China | Group 1-12 | Near-infrared (NIR) imaging system | (69/25)/(74/20) | (60.04±10.51)/(60.47±9.92) | Fujian Medical  University Union Hospital and Qinghai University affiliated Hospital | (43.6±18.4)/(46.3±22.8) | - | - |
| 2020-Yuan Tian et al. | China | Group 1-12 | Near-infrared (NIR) imaging system | (14/13)/(14/18) | (57.67±10.48)/(58.59±12.14) | Third Surgery Department of  the Fourth Hospital of Hebei Medical University | (29.3±8.8)/(28.4±12.8) | 23.76±2.91 | 23.52±2.85 |
| 2020-Xiaofeng Lu et al. | China | Group 1-12 | Near-infrared (NIR) imaging system | (19/9)/(20/8) | (57.96±12.66)/(59.17±9.17) | Nanjing Drum Tower Hospital, the Affiliated Hospital of Nanjing University Medical School | - | 22.25±2.32 | 22.86±2.73 |

**Table S2** Safety of laparoscopic gastrectomy of included studies about time of operation related to laparoscopic gastrectomy with indocyanine green**.**

| Year-Author | Country | Time of operation | | | |
| --- | --- | --- | --- | --- | --- |
|  |  | Number of participants in ICG group | Mean (SD) of time of operation in ICG group(min) | Number of participants in non-ICG group | Mean (SD) of time of operation in non-ICG group(min) |
| 2017-Yuan-Tzu Lan | China | 14 | 327±79.7 | 65 | 349.8±120.9 |
| 2019-Shuai Ma | China | 38 | 172.8±45.8 | 44 | 162.6±45.7 |
| 2020-Shin Hoo Park | Korea | 20 | 228.85±33.17 | 60 | 213.87±38.48 |
| 2018-Yuki Ushimaru | Japan | 84 | 206.1±5 | 84 | 237±5 |
| 2019-Ruhong Tu | China | 39 | 173±28 | 663 | 174±41 |
| 2020-Maoxing Liu | China | 61 | 207.21±33.63 | 75 | 239.25±44.19 |
| 2018-InGyu Kwon | American | 40 | 191±39.5 | 40 | 209±55.3 |
| 2019-Fabio Cianchi | Italy | 37 | 293.1±61 | 37 | 321.2±77.8 |
| 2020-Yuan Tian et al. | China | 27 | 230.52±20.71 | 32 | 238.78±34.44 |
| 2020-Xiaofeng Lu et al. | China | 28 | 260.18±46.7 | 28 | 277.86±69.15 |

**Table S3** Safety of laparoscopic gastrectomy of included studies about intraoperative blood loss related to laparoscopic gastrectomy with indocyanine green.

| Year-Author | Country | Intraoperative blood loss | | | |
| --- | --- | --- | --- | --- | --- |
|  |  | Number of participants in ICG group | Mean (SD) of intraoperative blood loss in ICG group | Number of participants in non-ICG group | Mean (SD) of intraoperative blood loss in non-ICG group |
| 2017-Yuan-Tzu Lan | China | 14 | 75.7±96.7 | 65 | 78.3±79.8 |
| 2019-Shuai Ma | China | 38 | 80.1±91.9 | 44 | 78.6±89.8 |
| 2020-Shin Hoo Park | Korea | 20 | 82.65±57.88 | 60 | 124.02±82.15 |
| 2018-Yuki Ushimaru | Japan | 84 | 10.1±6.6 | 84 | 36.9±6.6 |
| 2019-Ruhong Tu | China | 39 | 40±48.75 | 663 | 50±437.5 |
| 2020-Maoxing Liu | China | 61 | 38.2±18.03 | 75 | 49.87±30.73 |
| 2018-InGyu Kwon | American | 40 | 46.8±35.3 | 40 | 47.9±42.1 |
| 2020-Yuan Tian et al. | China | 27 | 40.19±18.21 | 32 | 44.69±13.73 |
| 2020-Xiaofeng Lu et al. | China | 28 | 144.64±83.15 | 28 | 167.5±141.23 |

**Table S4** Safety of laparoscopic gastrectomy of included studies about time of postoperative hospitalization related to laparoscopic gastrectomy with indocyanine green.

| Year-Author | Country | Time of postoperative hospitalization | | | |
| --- | --- | --- | --- | --- | --- |
|  |  | **Number of participants in ICG group** | **Mean (SD) of time of postoperative hospitalization in ICG group** | **Number of participants in non-ICG group** | **Mean (SD) of time of postoperative hospitalization in non-ICG group** |
| 2017-Yuan-Tzu Lan | China | 14 | 10.1±3.5 | 65 | 11.9±12.8 |
| 2019-Shuai Ma | China | 38 | 7±2 | 44 | 7.5±2.4 |
| 2020-Shin Hoo Park | Korea | 20 | 9.95±3.58 | 60 | 11.2±8.12 |
| 2018-Yuki Ushimaru | Japan | 84 | 7.5±0.1 | 84 | 8.7±0.1 |
| 2019-Ruhong Tu | China | 39 | 8.6±3.6 | 663 | 9.4±5 |
| 2020-Maoxing Liu | China | 61 | 7.95±1.99 | 75 | 8.23±5.12 |
| 2018-InGyu Kwon | American | 40 | 6.3±3.1 | 40 | 7.2±9.7 |
| 2019-Fabio Cianchi | Italy | 37 | 10±3.8 | 37 | 10.9±3.8 |
| 2020-Yuan Tian et al. | China | 27 | 8.30±3.64 | 32 | 8.22±3.63 |
| 2020-Xiaofeng Lu et al. | China | 28 | 13.46±8.92 | 28 | 15.71±9.15 |

**Table S5** Detailed of Indocyanine green.

| Year-Author | Country | The concentration of ICG(g/L) | Injection site of Indocyanine green | Injection timing of indocyanine green | ICG usage |
| --- | --- | --- | --- | --- | --- |
| 2017-Yuan-Tzu Lan et al. | China | 2.5 | Subserosa: 9  Submucosal:6 | ICG injection include intraoperative subserosa and preoperative submucosal injections surrounding the tumor | ICG (Taiwan Sankyo Pharmaceutical Co., Ltd) in a volume of 10 mL (25 mg in total) was gently injected at four injection sites (0.6 mL in each injection site) around the primary tumor. Approximately 1.5 mg of ICG was injected at each site. |
| 2019- Ma et al. | China | 1.25 | Submucosal | Injection of ICG under gastroscope 12 hour before operation | ICG was diluted with normal saline and prepared at a concentration of 1.25mg/ml solution was injected. The injection points were evenly distributed from the mouth to the anal side. The injection points avoided the tumor and 0.5ml ICG solution was injected at each point. |
| 2020-Shin‑Hoo Park et al. | Korea | 0.1 | Submucosal | Intraoperative submucosal injection of ICG (0.1 mg/mL) | A high-concentration solution (2.5 mg/mL) of ICG (Daiichi Sankyo Pharmaceutical Co., Ltd., Osaka, Japan) was prepared and then diluted with 0.9% normal saline to a concentration of 0.1 mg/mL. Intraoperatively, a small volume of the diluted ICG solution (1 mL, 0.1 mg/mL) was injected into the submucosa at five locations (on the lesser curvature side—low body and antrum; on the greater curvature side—mid body, low body and antrum) using a 23-gauge injection needle (Olympus Co., Ltd., Tokyo, Japan). |
| 2020-Ma et al. | China | 1.25 | Submucosal | Within 12h before surgery | ICG:1.25mg/ml; Method: endoscopic injection; Volume:0.5ml ICG solution per site; 3 points in lesser curvature; 4 points in greater curvature |
| 2020-Chen et al. | China | 1.25 | Submucosal | ICG to the submucosa 1 day before surgery. | The ICG powder (Dandong Yichuang Pharmaceutical Co) wasdissolvedin1.25 mg/mL of sterile water. Approximately 0.5 mL of the prepared solution, containing 0.625mg of ICG, was injected along the submucosa of the stomach at 4 points around the primary tumor, for a total volume of 2 mL |
| 2018-Yuki Ushimaru et al. | Japan | 0.05 | Submucosal | ICG group underwent GIF 1 day preoperatively | The solution for ICG marking was prepared by dissolving Diagnogreen 1 V in 10 ml of distilled water; 0.2 ml of this solution was mixed with 9.8 ml of distilled water to obtain 10 ml of solution (0.05 mg/ml of Diagnogreen). The prepared solution (0.5 ml at a time) was submucosally injected under endoscopic guidance at four sites around the tumor (proximal side, distal side, and bilateral sides) |
| 2019-Tu et al. | China | 1.25 | Submucosal | 1 day (20–30 hours) before surgery | Under conventional gastroscope in tumor primary tumors around 3, 6, 9, 12 point 4 in submucosal injection of ICG 0 - 6 mL (dandong medical and pharmaceutical co., LTD. Product, 25 mg/a). The ICG solution preparation: injection with ICG with sterilized injection diluted into 1.25 g/L |
| 2020-Liu et al. | China | 0.625 | Submucosal | Preoperative 24 hour | ICG (Dandong Yichuang Pharmaceutical Co., Dandong, China) solution was injected into the submucosa layer 1 day (20–30 hours) before surgery by the endoscopist. Four points (proximal, distal, and bilateral to the tumor region) were selected and 0.5 mL of ICG, diluted to 0.625 mg/mL, was injected into each point |
| 2018-InGyu Kwon et al. | American | 1.25 | Submucosal | 1 day before surgery | As a fluorescent contrast agent, ICG (Dongindang Pharmaceutical) was prepared as a 1.25-mg/mL solution in sterile water (Figure 1A). During endoscopy for tumor localization on the day before surgery,^16^ we injected 0.6 mL of the prepared solution, containing0.75mgofICG, along the submucosal layer of the stomach at4 points around the primary tumor. The total volume of injected ICG solution was 2.4 mL, for a total ICG amount of 3 mg. |
| 2019-Fabio Cianchi et al. | Italy | 1.25 | Submucosal | 1 day before surgery. | A 0.2% (1.25 mg/ml) ICG solution was gently injected into the submucosa layer with approximately 0.5 ml into the four quadrants around the tumor under endoscopic examination. |
| 2021-Zening Huang et al. | China | 0.5 | Subserosa | 1 day before surgery. | After preoperative exploration, ICG powder was dissolved in 0.5 mg/mL of sterile water, and the prepared solution (1.5 mL for each point) was injected along the subserosa of the stomach at six specifc points along the lesser and greater curvatures of the stomach. |
| 2020-Yuan Tian et al. | China | 2.5 | Submucosal | 1 day before surgery. | ICG was marked in the endoscopy division 1 day before surgery and injected submucosally at 4 points (proximal side, distal side, and left  and right sides) 0.5–1 cm from the tumor edge under endoscopy. The test dose for each point was approximately 0.5 ml. |
| 2020-Xiaofeng Lu et al. | China | 2.5 | Submucosal | 1 day before surgery. | Endoscopic ICG injection intraoperatively. The ICG powder was diluted to 2.5mg/ml and the prepared solution (0.5ml at a time) was injected at proximal and distal submucosa of the tumor. |

**Table S6** Characteristics of patients of included studies.

| Year-Author | Country | Pathological T category in ICG group | Pathological T category in non-ICG group | Types of gastrectomy in ICG group | Types of gastrectomy in non-ICG group | TNM stage(Ⅰ/Ⅱ/Ⅲ) of ICG group | TNM stage(Ⅰ/Ⅱ/Ⅲ) of non-ICG group | Guideline of TNM stage | Pathological type |
| --- | --- | --- | --- | --- | --- | --- | --- | --- | --- |
| 2017-Yuan-Tzu Lan et al. | China | 8:3:1:2 (T1:T2:T3:T4) | 47:7:9:2 (T1:T2:T3:T4) | Radical subtotal gastrectomy：11 radical total gastrectomy：3 | Radical subtotal gastrectomy：59； radical total gastrectomy：6 | 8/2/4 | 43/15/7 | AJCC cancer staging manual (7th ed). | Adenocarcinoma |
| 2019- Ma et al. | China | 13:25 (T1-2:T3-4) | 16/28 (T1-2:T3-4) | Distal gastrectomy：30 total gastrectomy：4 proximal gastrectomy:4 | Distal gastrectomy：31 total gastrectomy：6 proximal gastrectomy:7 | 18/20 (Ⅰ-Ⅱ/Ⅲ) | 18/26 (Ⅰ-Ⅱ/Ⅲ) | AJCC cancer staging manual (8th ed). | Adenocarcinoma |
| 2020-Shin‑Hoo Park et al. | Korea | 18:2 (T1:T2-T4) | 59:1 (T1:T2-T4) | Distal gastrectomy | Distal gastrectomy | 17/3 (Ⅰ/Ⅱ-Ⅳ) | 58/2 (Ⅰ/Ⅱ-Ⅳ) | AJCC cancer staging manual (7th ed). | Adenocarcinoma |
| 2020-Ma et al. | China | - | - | Distal Gastrectomy | Distal Gastrectomy | - | - | - | - |
| 2020-Chen et al. | China | 35:68:26 (T1:T2-3:T4a) | 29:70:30 (T1:T2-3:T4a) | Distal gastrectomy：71 Total gastrectomy：58 | Distal gastrectomy：43 Total gastrectomy：86 | 50/33/46 | 41/33/55 | AJCC cancer staging manual (8th ed). | Adenocarcinoma |
| 2018-Yuki Ushimaru et al. | Japan | 55:12:11:6 (T1:T2:T3:T4) | 59:5:15:5 (T1:T2:T3:T4) | Distal gastrectomy | Distal gastrectomy：80 Total gastrectomy：4 | 60/17/7 | 60/12/12 | Japanese Gastric Cancer A. Japanese classification of gastric carcinoma: 3rd English edition. | Adenocarcinoma |
| 2019-Tu et al. | China | 9：8：18：4：0 (T1：2：3：4a:4b） | 198:73:238:149:5 (T1：2：3：4a:4b） | Distal gastrectomy：16 total gastrectomy：21 proximal gastrectomy:2 | - | 9:5:3:2:12:4:4 (IA/IB/IIA/IIAB/IIIA/IIIB/IIIC) | 168:57:90:81:108:92:67 (IA/IB/IIA/IIAB/IIIA/IIIB/IIIC) | AJCC cancer staging manual (8th ed). | Adenocarcinoma or signet ring cell carcinoma |
| 2020-Liu et al. | China | 30:19:7:5 (T1:T2:T3:T4) | 38:20:12:5 (T1:T2:T3:T4) | Distal gastrectomy | Distal gastrectomy | 37/18/6 | 44/20/21 | AJCC cancer staging manual (7th ed). | - |
| 2018-InGyu Kwon et al. | American | 24:11:5 (T1a:T1b:T2) | 25:11:4 (T1a:T1b:T2) | Distal gastrectomy：34 total gastrectomy：6 | Distal gastrectomy：32 total gastrectomy：8 | All of them were I(T1N0M0,T1N1M0,andT2N0M0) | All of them were I(T1N0M0,T1N1M0,andT2N0M0) | AJCC cancer staging manual (7th ed). | Adenocarcinoma |
| 2019-Fabio Cianchi et al. | Italy | 6:11:20 (T1:T2:T3) | 7:5:25 (T1:T2:T3) | Radical subtotal gastrectomy：25； radical total gastrectomy：12 | Radical subtotal gastrectomy：30； radical total gastrectomy：7 | 12/5/20 | 12/9/16 | AJCC cancer staging manual (7th ed). | - |
| 2021-Zening Huang et al. | China | 11:33:26:24 (T1:T2:T3:T4) | 11:28:27:28 (T1:T2:T3:T4) | Distal gastrectomy：79 total gastrectomy：15 | Distal gastrectomy：82 total gastrectomy：12 | 26/44/24 | 27/38/29 | AJCC cancer staging manual (7th ed). | Adenocarcinoma |
| 2020-Yuan Tian et al. | China | 17:8:3:0 (T1:T2:T3:T4) | 19:5:3:1 (T1:T2:T3:T4) | Proximal gastrectomy:3 Distal gastrectomy：10  total gastrectomy：15 | Proximal gastrectomy:6 Distal gastrectomy:13  total gastrectomy: 9 | 225/3  (Ⅰ-Ⅱ/Ⅲ-Ⅳ) | 24/4  (Ⅰ-Ⅱ/Ⅲ-Ⅳ) | Japanese Gastric Cancer A. Japanese classification of gastric carcinoma: 3rd English edition. | Adenocarcinoma |
| 2020-Xiaofeng Lu et al. | China | 13:7:7 (T1:T2:T3) | 10:14:8 (T1:T2:T3) | Distal gastrectomy | Distal gastrectomy | 12/9/6 | 12/13/7 | AJCC cancer staging manual (7th ed). | Adenocarcinoma |

**Table S7** Tumor location of patients (1).

| Year-Author | Country | Tumor location of ICG group (longitudinal) | | | | | Tumor location of non-ICG group(longitudinal) | | | | |
| --- | --- | --- | --- | --- | --- | --- | --- | --- | --- | --- | --- |
|  |  | **High body** | **mid body** | **Low body** | **Angle** | **Antrum** | **High body** | **Mid body** | **Low body** | **Angle** | **Antrum** |
| 2017-Yuan-Tzu Lan et al. | China | 2 | 1 | 3 | 5 | 3 | - | - | - | - | - |
| 2019- Ma et al. | China | - | - | - | - | - | - | - | - | - | - |
| 2020-Shin‑Hoo Park et al. | Korea | - | - | 2 | 4 | 14 | - | - | 5 | 11 | 44 |
| 2020-Ma et al. | China | - | - | - | - | - | - | - | - | - | - |
| 2020-Chen et al. | China | 33 | 21 | 75 | - | - | 66 | 14 | 49 | - | - |
| 2018-Yuki Ushimaru et al. | Japan | - | 52 | 32 | - | - | - | 55 | 29 | - | - |
| 2019-Tu et al. | China | 15 | 8 | 15 | - | - | 207 | 126 | 296 | - | - |
| 2020-Liu et al. | China | - | 23 | 38 | - | - | - | 26 | 49 | - | - |
| 2018-InGyu Kwon et al. | American | 4 | 16 | 20 | - | - | 8 | 13 | 19 | - | - |
| 2019-Fabio Cianchi et al. | Italy | 6 | 11 | 20 | - | - | 4 | 10 | 23 | - | - |
| 2021-Zening Huang et al. | China | 41 | 22 | 17 | - | - | 38 | 30 | 14 | - | - |
| 2020-Yuan Tian et al. | China | 7 | 11 | 10 | - | - | 10 | 5 | 13 | - | - |
| 2020-Xiaofeng Lu et al. | China |  |  |  | 12 | 9 |  |  |  | 11 | 14 |

**Table S7** Tumor location of patients (2).

| Year-Author | Country | Tumor location of ICG group (circumferential) | | | | | Tumor location of non-ICG group(circumferential) | | | | |
| --- | --- | --- | --- | --- | --- | --- | --- | --- | --- | --- | --- |
|  |  | **Lesser curvature** | **Greater curvature** | **Anterior wall** | **Posterior wall** | **Encircling involvement** | **Lesser curvature** | **Greater curvature** | **Anterior wall** | **Posterior wall** | **Encircling involvement** |
| 2017-Yuan-Tzu Lan et al. | China | 6 | 1 | 3 | 4 | 0 | - | - | - | - | - |
| 2019- Ma et al. | China | - | - | - | - | - | - | - | - | - | - |
| 2020-Shin‑Hoo Park et al. | Korea | 6 | 7 | 4 | 3 | 0 | 26 | 8 | 14 | 12 | 0 |
| 2020-Ma et al. | China | - | - | - | - | - | - | - | - | - | - |
| 2020-Chen et al. | China | - | - | - | - | - | - | - | - | - | - |
| 2018-Yuki Ushimaru et al. | Japan | 37 | 13 | 15 | 17 | 2 | 41 | 11 | 14 | 17 | 1 |
| 2019-Tu et al. | China | - | - | - | - | - | - | - | - | - | - |
| 2020-Liu et al. | China | - | - | - | - | - | - | - | - | - | - |
| 2018-InGyu Kwon et al. | American | - | - | - | - | - | - | - | - | - | - |
| 2019-Fabio Cianchi et al. | Italy | - | - | - | - | - | - | - | - | - | - |
| 2021-Zening Huang et al. | China | - | - | - | - | - | - | - | - | - | - |
| 2020-Yuan Tian et al. | China | - | - | - | - | - | - | - | - | - | - |
| 2020-Xiaofeng Lu et al. | China | - | - | - | - | - | - | - | - | - | - |
